# Combination of Extended Antivirals With Antiretrovirals for Severe Mpox in Advanced Human Immunodeficiency Virus Infection: Case Series of 4 Patients

**DOI:** 10.1093/ofid/ofae110

**Published:** 2024-02-27

**Authors:** Michael T Duong, Pablo Tebas, Bhavya Ancha, Jillian Baron, Pallavi Chary, Stuart N Isaacs, Zsofia Szep

**Affiliations:** Division of Infectious Diseases, Department of Medicine, Perelman School of Medicine, University of Pennsylvania, Philadelphia, Pennsylvania, USA; Division of Infectious Diseases, Department of Medicine, Perelman School of Medicine, University of Pennsylvania, Philadelphia, Pennsylvania, USA; Division of Infectious Diseases, Department of Medicine, Perelman School of Medicine, University of Pennsylvania, Philadelphia, Pennsylvania, USA; Division of Infectious Diseases, Department of Medicine, Perelman School of Medicine, University of Pennsylvania, Philadelphia, Pennsylvania, USA; Division of Infectious Diseases, Department of Medicine, Perelman School of Medicine, University of Pennsylvania, Philadelphia, Pennsylvania, USA; Division of Infectious Diseases, Department of Medicine, Perelman School of Medicine, University of Pennsylvania, Philadelphia, Pennsylvania, USA; Division of Infectious Diseases, Department of Medicine, Corporal Michael J. Crescenz Veterans Affairs Medical Center, Philadelphia, Pennsylvania, USA; Division of Infectious Diseases, Department of Medicine, Perelman School of Medicine, University of Pennsylvania, Philadelphia, Pennsylvania, USA; Division of Infectious Diseases, Department of Medicine, Corporal Michael J. Crescenz Veterans Affairs Medical Center, Philadelphia, Pennsylvania, USA

**Keywords:** mpox, HIV, antiviral, tecovirimat, cidofovir

## Abstract

To gauge the safety and utility of extended tecovirimat/cidofovir for severe mpox, here we report our experience caring for 4 patients with mpox and advanced human immunodeficiency virus (HIV) at the Hospitals of the University of Pennsylvania during the 2022 global outbreak. Three patients had recurrent courses complicated by superinfections, coinfections and insufficient nutrition/housing, requiring extended tecovirimat (5–16 weeks) and cidofovir (1–12 doses) with probenecid and fluids. At follow-up, patients had undetectable HIV RNA on antiretrovirals, improved ulcers and stable renal function on antivirals. Serology guided cessation for one 7-month cidofovir course. Overall findings support a comprehensive approach of prolonged tecovirimat/cidofovir with antiretrovirals for severe mpox, while addressing social factors.

Mpox is an orthopoxvirus related to the now-eradicated smallpox. While mpox was endemic to Central/West Africa [1], a global mpox outbreak began in May 2022, culminating in a World Health Organization health emergency declaration [2]. During the 2022–2023 outbreak, several studies suggested that about half of people with mpox had concomitant human immunodeficiency virus (HIV) infection [3–5]. Notably, some cohorts had a coinfection rate of 70%, suggesting the role of geographic heterogeneity in clinical presentation [3]. Of patients with HIV and mpox, those with advanced HIV are associated with high morbidity and mortality while those with HIV suppressed on antiretroviral therapy (ART) and high CD4 cell count have presentations comparable to people without HIV [5–7].

Treatment for mpox is currently limited given that no drugs have US Food and Drug Administration (FDA) approval for mpox. While multiple reports have investigated assorted antivirals in patients with mpox, a subset of whom have HIV [4, 5, 8–13], treatment of mpox and advanced HIV disease is mostly based on anecdotal experience as clinical trials are limited. Here, we present a case series of patients with severe mpox and advanced HIV treated with a combination of extended anti-mpox therapy (tecovirimat and cidofovir) with ART.

Tecovirimat was FDA-approved in 2018 for smallpox [14, 15] and made available for mpox through an expanded-access investigational new drug application held by the Centers for Disease Control and Prevention (CDC). Tecovirimat targets viral protein p37 involved in cell-to-cell viral transmission but does not block viral replication, so additional therapies may be required to completely eradicate mpox in severely immunocompromised patients [16]. Moreover, resistance to tecovirimat can occur with single mutations. Tecovirimat is taken intravenously or orally, the latter requiring a fatty meal for optimal bioavailability [17].

Cidofovir was first approved for cytomegalovirus retinitis. Once phosphorylated, cidofovir is incorporated into viral DNA, triggering chain termination. Cidofovir has activity against poxviruses [18, 19]. Yet, cidofovir is associated with significant nephrotoxicity via accumulation in the proximal tubules. To address this, cidofovir is administered with intravenous fluids and probenecid, which inhibits organic anion transporters in the nephron. Brincidofovir, an oral prodrug of cidofovir, was developed as another therapeutic against smallpox [20]. However, due to the unavailability of the drug and logistical challenges, it was not widely used during the 2022–2023 mpox outbreak [19].

Initial recommendations supported tecovirimat as mpox monotherapy [15]. About 20% of the patients with mpox and HIV reported in 1 global case series received tecovirimat and only 2% received a combination of anti-mpox antivirals [6]. Some studies have also cautioned about initiating ART during mpox infection due to risk of immune reconstitution inflammatory syndrome (IRIS).

However, this monotherapy approach presents challenges for patients with advanced HIV as tecovirimat relies on the immune system to clear the virus. Thus, after stopping a standard 14-day course of tecovirimat, mpox lesions would often grow or reappear. Moreover, prolonged monotherapy creates an ideal environment to develop resistance, which can lead to treatment failure [6, 15, 21]. Our case series aims to describe our experience with the safety and efficacy of extended tecovirimat, cidofovir, and ART in patients with severe mpox and advanced HIV infections.

## METHODS

The authors are members of the infectious diseases group at the Hospitals of the University of Pennsylvania (Penn). The 4 patients with advanced HIV and mpox reported here were under the care of the authors. We were unaware of other patients at Penn with advanced HIV and mpox during this period. Relevant data and clinical courses were collected by retrospective chart review (Table 1). Laboratory studies, including HIV-1 RNA load quantification and CD4^+^ T-cell counts, were obtained from hospital records at Penn via electronic health record search. Mpox diagnosis was made by orthopox non-variola positivity on polymerase chain reaction (PCR). Corneal involvement with mpox keratitis was corroborated by fluorescein stain images (Supplementary Figure 1). Our study did not include factors requiring patient consent and was determined to not be research requiring approval by Penn's institutional review board.

**Table 1.** Summary of Clinical Cases With Mpox and Advanced Human Immunodeficiency Virus at the Hospitals of the University of Pennsylvania

Case	Age and Sex	Presentation and Factors Affecting Recurrence	HIV RNA Load and CD4 Count From Start → End	Mpox Treatment	HIV Treatment
1	40s, M	Recurrent mpox ulcers with keratitis and conjunctival lesions due to inadequate caloric intake for PO absorption and ART. HSV-1/2–positive.	RNA: 43 100 → 65 copies/mL CD4: 53 → 61 cells/μL	PO/IV tecovirimat (16 wk), IV cidofovir (9 doses, 4 mo), GTT trifluridine (3 wk)	BIC/FTC/TAF with DRV/COBI → DOR
2	50s, M	Recurrent mpox lesions related to inconsistent housing and wound care. MRSA–positive.	RNA: 2104 → 138 copies/mL CD4: 41 → 71 cells/μL	PO tecovirimat (5 wk, then lost to follow-up), partial IV cidofovir (1 dose)	BIC/FTC/TAF
3	30s, M	Recurrent mpox ulcers from difficulties in caloric intake, adherence, and ART management.	RNA: 0 → 0 copies/mLCD4: <35 → 65 cells/μL	PO/IV tecovirimat (8 wk), IV cidofovir (12+ doses, 7 mo)	BIC/FTC/TAF → CBG/RPV and LEN
4	30s, M	Multiple mpox skin lesions and dysphagia. No recurrences. SARS-CoV-2 positive.	RNA: 217 000 → 0 copies/mL CD4: 0 → 83 cells/μL	PO tecovirimat (6 wk), IV cidofovir (6 doses, 3 mo)	BIC/FTC/TAF

Abbreviations: ART, antiretroviral therapy; BIC/FTC/TAF, bictegravir/emtricitabine/tenofovir alafenamide; CBG/RPV, cabotegravir/rilpivirine; SARS-CoV-2, severe acute respiratory syndrome coronavirus 2; DOR, doravirine; DRV/COBI, darunavir/cobicistat; GTT, eye drops; HIV, human immunodeficiency virus; HSV, herpes simplex virus; IV, intravenous; LEN, lenacapavir; M, male; MRSA, methicillin-resistant *Staphylococcus aureus*; PO, oral.

## CASE PRESENTATIONS

### Case 1: Severe Mpox With Keratitis Requiring Multiple Hospitalizations and Extended Antivirals

In late 2022, a man in his 40s, with history of advanced HIV since 2005 and poor adherence to ART, experienced multiple admissions for severe mpox infection characterized by deteriorating skin and ophthalmic lesions, despite treatment with tecovirimat and cidofovir.

Initially, he was admitted with oral ulcers following dental extraction, subsequently progressing to tender, necrotic lesions across his face, arms, and chest that were mpox–positive. He began oral tecovirimat (600 mg twice daily) and received 1 dose of intravenous (IV) cidofovir (5 mg/kg) with probenecid (500 mg) and IV hydration, restarting bictegravir/emtricitabine/tenofovir alafenamide before discharge to complete 2 weeks of oral tecovirimat. His wounds improved substantially, displaying crusting and granulation tissue.

After completing tecovirimat, 3 weeks later, he was readmitted with worsening body ulcers and bilateral corneal lesions accompanied by conjunctivitis. Eye swabs demonstrated herpes simplex virus (HSV)–1/2 by PCR. CD4 count remained low (<35 cells/μL) and HIV RNA was 194 000 copies/mL. A switch to doravirine-based ART was guided by resistance testing. IV tecovirimat (200 mg twice daily) and cidofovir (5 mg/kg) were initiated, with corneal ulcers managed via valacyclovir (1 g every 8 hours) and ophthalmic trifluridine (1% solution, 1 drop every 3 hours). He improved and was discharged with oral tecovirimat (600 mg twice daily) and IV cidofovir (every 2 weeks with probenecid/fluids).

During his third hospital admission, 2 months after initial presentation, he exhibited worsening mpox skin and eye lesions (Supplementary Figure 1). Ophthalmic assessment revealed keratitis and conjunctival ulcers, with positive swabs for mpox and *Arcanobacterium*. Despite improved HIV control (HIV RNA, 65 copies/mL; CD4 count, 61 cells/μL), his condition warranted resumption of IV tecovirimat (200 mg twice daily), cidofovir (5 mg/kg), ophthalmic trifluridine (1% solution), and valacyclovir (1 g every 8 hours). Subsequent improvement led to resolution of conjunctival ulcers and keratitis by time of discharge. He continued oral tecovirimat (600 mg twice daily for a total of 16 weeks on tecovirimat), ophthalmic trifluridine (for 3 weeks), and cidofovir (5 mg/kg IV every 2 weeks over several months for a total of 9 doses). Renal function was stable throughout his treatment course.

At follow-up 4 months after initial presentation, substantial improvement was evident, with most of his skin wounds healed and no active ocular lesions. HIV control had improved (CD4 count, 115 cells/μL; HIV RNA, <40 copies/mL), though persistent mild edema and scarring remained in 1 leg.

### Case 2: Severe Mpox Exacerbated by Inconsistent Housing and Wound Care

In late 2022, a man in his 50s with advanced HIV was admitted to Penn displaying severe mpox skin lesions. The patient's mpox symptoms first appeared during his time in prison when the initial small sores were believed to be varicella zoster. The diffuse lesions continued to enlarge and tested positive for mpox. He experienced raised lesions on his face and mouth, which later spread to his torso and hands. After admission to our hospital, he began a 14-day course of oral tecovirimat (600 mg twice daily) and reinitiated bictegravir/emtricitabine/tenofovir alafenamide. Though he could only complete 8 days of the intended 14-day course, his lesions began improving with granulation tissue formation.

One month later, the lesions reappeared, leading to his admission at an outside hospital where he received IV tecovirimat and vancomycin due to methicillin-resistant *Staphylococcus aureus* (MRSA) superinfection. HIV RNA was 2104 copies/mL and CD4 count 41 cells/μL. After leaving by self-directed discharge, he arrived at Penn, where IV cidofovir (5 mg/kg with fluids/probenecid) was added to attenuate concerns of tecovirimat resistance. The plan included scheduled outpatient cidofovir infusions and transitioning to oral tecovirimat. However, he opted for self-discharge and continued only oral tecovirimat and ART, missing scheduled cidofovir infusions.

He was treated for superinfection of previous lesions with oral doxycycline 1 month later, related to inconsistent wound care appointments. During this period, he had improved adherence to ART (CD4 count, 71 cells/μL; HIV RNA, 138 copies/mL) and oral tecovirimat, ensuring adequate caloric intake. Unfortunately, he faced housing instability and difficulties in accessing wound care and coming to Penn for cidofovir infusions. He was consequently lost to follow-up, leaving his medical progress uncertain.

### Case 3: Severe Mpox With Extended Antivirals Guided by Serology

A male patient in his 30s with HIV, off ART and out of care for at least a year, presented to an outside hospital emergency department in fall 2022 with facial lesions. Orthopoxvirus PCR testing in the emergency room of a facial lesion was positive and he was started on tecovirimat 600 mg every 12 hours for 14 days. He was linked back to care for management of HIV and mpox, at which time his CD4 count was <35 cells/μL. He was restarted on his prior ART regimen with bictegravir/emtricitabine/tenofovir alafenamide and darunavir/cobicistat, but had difficulty with adherence. His facial lesions initially crusted over; however, within a week of stopping tecovirimat, facial lesions began to expand and new ulcerations began to appear on his face and extremities. He was restarted on tecovirimat with a higher dose of 600 mg every 8 hours due to his weight of 120 kg. He was initially planned for an additional 14-day course but continued to develop new lesions while on treatment. During this time he had ongoing immunosuppression with CD4 count <50 cells/μL and food insecurity leading to inability to take tecovirimat with food. After 4 weeks of high-dose tecovirimat, the lesions on his face and head continued to expand with the appearance of new ulcerations, and he developed a new ulceration with edema and erythema on his left foot, limiting his ability to walk.

Given progression, he was admitted to the hospital and started on tecovirimat 600 mg IV every 12 hours and cidofovir 5 mg/kg IV with initial loading dose week 1, followed by maintenance dosing every 2 weeks starting week 2. On this regimen, he exhibited skin healing and was discharged to continue oral tecovirimat (600 mg every 12 hours) and IV cidofovir (5 mg/kg every 2 weeks maintenance dosing), in addition to bictegravir/emtricitabine/tenofovir alafenamide and darunavir/cobicistat ART.

The plan was to continue every 2 weeks cidofovir infusions and oral tecovirimat until CD4 count was >200 cells/μL. Yet with ongoing difficulty taking multiple medications, tecovirimat was stopped after 2 months to focus on ART adherence. He continued cidofovir infusions every 2 weeks for >7 months while awaiting immune reconstitution. However, given continued challenges with oral medications, he started on injectable cabotegravir-rilpivirine with lenacapavir. On an injectable regimen, he maintained HIV suppression and CD4 reached >100 cells/μL. CDC serologic testing for orthopoxvirus demonstrated immunoglobulin G (IgG)–positive and immunoglobulin M–negative mpox titers when CD4 count was 67 cells/μL. Due to difficulty with the patient attending further infusion appointments, cidofovir infusion was stopped and no further lesions developed. Mpox vaccination was held given IgG–positive mpox serology.

### Case 4: Full Recovery From Mpox After 1 Extended Course of Antivirals

A male in his 30s presented to our hospital in late 2022 with multiple painful oral and skin mpox lesions on his face and torso. He had severe oral intake limitation, dysphagia, and difficulty speaking. On admission, he was also diagnosed with HIV (CD4 count, 0 cells/μL; HIV RNA, 217 000 copies/mL) and severe acute respiratory syndrome coronavirus 2 (SARS-CoV-2) infections by PCR without pulmonary symptoms.

The patient started on fixed-dose combination of bictegravir/emtricitabine/tenofovir alafenamide as well as dual anti-mpox therapy. Because of his dysphagia and decreased ability to maintain a high-fat diet for optimal oral tecovirimat absorption, he was given oral tecovirimat (600 mg twice daily) and cidofovir (5 mg/kg IV with concomitant hydration and probenecid) per CDC guidelines [15]. He also received a 5-day course of IV remdesivir because of risk of developing severe coronavirus disease 2019. Following treatment, his oral intake and speech improved, and he was discharged on oral tecovirimat (600 mg twice daily for a total of 6 weeks) and 6 doses of cidofovir (5 mg/kg IV for 2 doses during first week, then every other week). Subsequently, skin ulcers resolved, but he developed depigmented scars. After 5 weeks, HIV RNA became undetectable and CD4 count increased markedly. His kidney function remained stable throughout the entire process.

## DISCUSSION

This case series describes our group's experience using extended cidofovir and tecovirimat in patients with severe mpox, advanced HIV, and complex social dynamics. All patients had improvement in mpox lesions, HIV RNA, and/or CD4 count. Moreover, our cases support that a prolonged cidofovir regimen with probenecid/fluids is effective in treating mpox without impairing renal function. Likewise, oral tecovirimat should be combined with daily nutrition with sufficient calories and fat for appropriate absorption. Prior reports also showed the efficacy of cidofovir in treating mpox [6, 19], and its incorporation into combined antivirals hold promise. Though generalization of these findings is needed, our report on these 4 patients with mpox ulcer recurrence, inadequate daily nutrition, and/or social factors influencing access to care may suggest that optimizing an extended regimen of dual anti-mpox therapies and daily nutrition was associated with lesion resolution. Despite the inherent complexity of administering combination antivirals, particularly cidofovir, this approach was well-tolerated and did not lead to nephrotoxicity.

Tecovirimat alone in a population with high lesion burden and mpox replication may lead to resistance, as has been suggested in some case series [6, 21]. Our cases concur with the current CDC recommendations [15] that now advocate for combination antivirals for individuals with advanced HIV and mpox from the onset of clinical presentation. Combined strategy mitigates emergence of resistance that may arise from tecovirimat monotherapy [6, 15, 21].

Our experiences attest to the importance of ART. Here, mpox and advanced HIV were managed concurrently, as all of our patients required ART while 2 patients needed ART adjustments. Benefits of HIV suppression outweighed potential risks of IRIS. While IRIS harbors potential of morbidity and mortality, especially intracranial infections, none of our patients experienced IRIS.

This case series illustrates substantial challenges posed by mpox in people with HIV, particularly those with low CD4 counts and uncontrolled HIV infection. We describe a spectrum of clinical manifestations, encompassing skin lesions on the face, trunk and extremities with mucosal, ocular and pelvic involvement. Additional factors that complicated their care included patient access to stable housing and wound care, prophylaxis, and coinfections such as SARS-CoV-2, HSV-1/2, and MRSA.

Our first case highlighted ophthalmic manifestations of mpox (Supplementary Figure 1). About 20% and 7% of patents with mpox exhibit conjunctivitis and keratitis, respectively [22]. Treatment with trifluridine and cidofovir is supported by several reports [22, 23]. Investigational vaccinia immunoglobulin intravenous treatment (VIGIV) was considered, but not pursued given response on tecovirimat and cidofovir and patient preference for nonparenteral therapy. Studies in rabbits suggested that VIGIV increased corneal scarring in viral keratitis [24], which further tempered our interest.

Our study had several limitations. This case series only had 4 patients from 1 care center and would benefit from a larger cohort. Long-term efficacy data were not available for our second case, due to social factors and loss to follow-up. Clinical recovery from people with mpox and advanced HIV can be protracted, with some courses spanning over 7 months. This extended timeframe for resolution informs patient expectations and management. Clinical presentations and circumstances were heterogeneous but did encompass several patients with tecovirimat and cidofovir regimens and severe mpox manifestations in the setting of advanced HIV. However, this case series does present preliminary evidence to suggest the role of extended, dual anti-mpox therapy with ART for patients with severe mpox and HIV.

Together, this retrospective report illustrates prolonged cidofovir with tecovirimat (over several weeks/months) as safe for managing severe mpox infections in people with advanced HIV. Our case series portrays multifaceted challenges including clinical manifestations and social determinants like housing stability, nutritional intake for oral tecovirimat absorption, and concurrent infections. Our findings advocate for comprehensive management integrating ART with the use of multiple anti-mpox antivirals, in addition to simultaneously addressing contextual and social factors. Our conclusions are limited by the retrospective and anecdotal nature of the case series. Nevertheless, there is a clear need for more research to better understand the utility of prolonged antiviral treatment targeting mpox in those with advanced HIV infection.

## Supplementary Material

ofae110_Supplementary_Data

## Supplementary Data

Supplementary materials are available at *Open Forum Infectious Diseases* online. Consisting of data provided by the authors to benefit the reader, the posted materials are not copyedited and are the sole responsibility of the authors, so questions or comments should be addressed to the corresponding author.

## Notes

***Patient consent.*** Our study does not include factors necessitating patient consent and was not considered to be research by Penn's institutional review board.

***Financial support*.** No reported financial support.
