# Supplementary material for: Combination of Extended Antivirals With Antiretrovirals for Severe Mpox in Advanced Human Immunodeficiency Virus Infection: Case Series of 4 Patients
Source: Open Forum Infect Dis. 2024 Feb 27;11(3):ofae110. doi: 10.1093/ofid/ofae110 (PMC10939438; doi:10.1093/ofid/ofae110)
Supplement: ofae110_Supplementary_Data [file ofae110_supplementary_data.docx]

**Supplementary Figures**


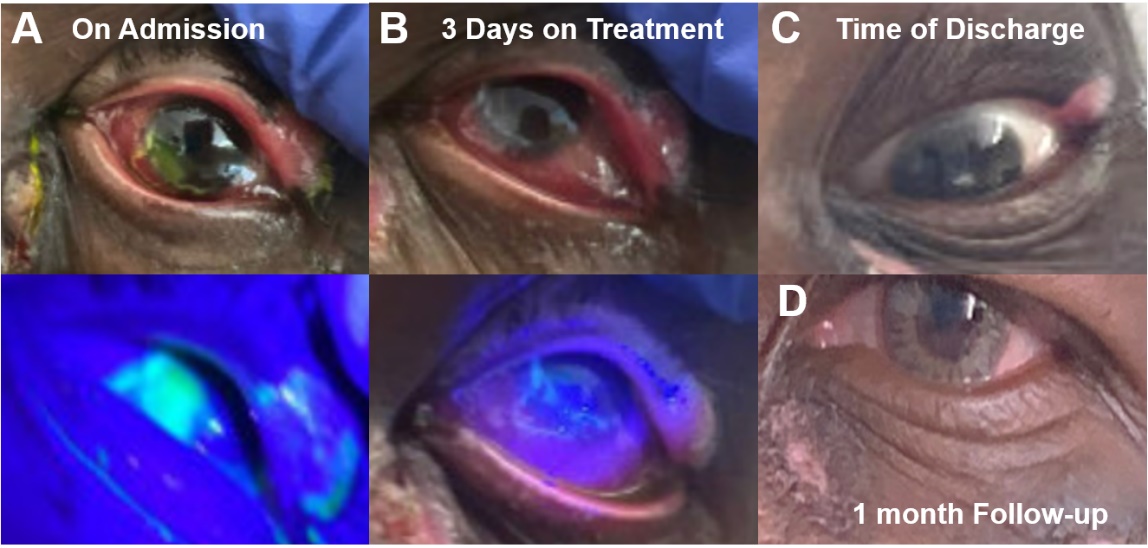


**Supplementary Figure 1**. Keratitis and conjunctivitis in a patient with mpox on (**A**) admission with mpox+ eye swab, (**B**) after 3 days of tecovirimat, trifluridine and acyclovir/valacyclovir (with fluorescein stain images at bottom), (**C**) at time of discharge, 11 days from admission and (**D**) at 1 month follow-up visit.
